# Adherence to Clinical Guidelines for Fibromyalgia: Help or Hindrance?

**DOI:** 10.1080/24740527.2023.2255070

**Published:** 2023-11-10

**Authors:** Winfried Häuser, Mary-Ann Fitzcharles

**Affiliations:** Department of Psychosomatic Medicine and Psychotherapy, Technische Universität München, München, Germany; Department of Rheumatology, McGill University, Montreal, Quebec, Canada; Alan Edwards Pain Management Unit, McGill University, Montreal, Quebec, Canada

Sir William Osler is quoted as saying, “The desire to take medicine is perhaps the greatest feature which distinguishes man from animals.” Drug treatment recommendations are today incorporated into clinical practice guidelines, intended to facilitate optimal patient care by providing guidance for the health care community and patients. Developed by a process of prespecified methodology, a group of experts examines the evidence-based literature and makes recommendations based on the available evidence and, when lacking, can provide expert input. Viewed as tools rather than rules, the uptake of guidelines by health care workers is often limited, with barriers ranging from social, political, and cultural factors to patient and health care provider factors.^1^ This poor adherence to guideline recommendation for the drug management of fibromyalgia (FM) is highlighted in the study by De Clifford-Faugere in this issue of the *Canadian Journal of Pain*.

Although the constellation of symptoms that constitute FM has been recognized since the 1880s, then termed neurasthenia, named “fibromyalgia” by Yunus et al. in 1981, and recognized as a defined disease entity by the American Medical Association in 1987, FM in this 21st century remains controversial.^2^ With diagnosis dependent on subjective symptom report, absence of a consistent examination finding, no biomarker, and, importantly, no “gold standard” of treatment, FM continues to challenge. Against this background, various groups have provided direction regarding ideal management of a condition that affects between 2% and 8% of the general population.^3–6^ The focus of clinical care is nonpharmacologic multimodal management, although this ideal is often not easily attainable, with patients and the health care community frequently turning to drug treatments, which have at best shown modest effect.

The study by De Clifford-Faugère et al. is noteworthy for two reasons: due to, firstly, deviation from guideline drug recommendations and, secondly, the very high use of multiple drugs by individual patients.^7^ In this small study of 63 FM participants, over half reported current use of five or more drugs to treat symptoms of FM, and 10% used an astounding ten or more drugs. Contrary to guideline recommendations, there was prevalent use of opioids in various forms/combinations (3.2%–23.8% with additive number of 79.4%), nonsteroidal anti-inflammatory drugs (60.3%), benzodiazepines/anxiolytics (34.9%), and cannabinoids (52.4%). Attention was paid to adverse effects but without information on perceived efficacy. In contrast to other recent large studies of drug use in FM, there was no control group, no information on previous FM treatments including nonpharmacologic strategies, or more precise information on current drug treatment.^8,9^ The deviation from guidelines is, however, not dissimilar from other larger studies in FM, or other illnesses, but the extremely high rate of medication use is concerning and requires understanding.

If these results are indeed valid and some patients are truly taking up to ten different medications for FM treatment, then where does the fault lie? Adherence can be perceived as bidirectional from the perspective of the health care provider and that of the patient. Physicians, especially those in primary care, may simply be overburdened and use a prescription as an easy solution to patient care, rather than the more time-intensive focus on nonpharmacological measures; alternatively, patients may be accessing care from multiple sources without sufficient disclosure. Because this survey^7^ was administered only in French, it is notable that the two guideline/algorithms used by the authors were available in French, so language cannot be used as a reason for nonadherence.^5,10^ Adherence to guidelines has advantages beyond simply good patient care and safety. Lower health care resource use and overall costs were reported for a retrospective claims database that included 150,321 patients with FM in the United States, even though FM adherence was only 33.5%.^11^

Accurate information in a study is critical to ensure the validity of findings and is reliant on the rigor of the study conduct and participant reliability. Though survey data provide insight into the real-world status of a condition, they are clearly open to biases. Who are the people who respond to survey requests? They are likely those with a specific interest in the condition but also with sufficient time to participate and, when applicable, with access to and familiarity with digital technology. Participant selection in the study by De Clifford-Faugère et al. may have influenced the results; they had previously participated in a web-based survey and then were preselected according to alphabetical order for this study.^7^ This created a sample unrepresentative of the general population because respondents were highly educated, with 63% reporting college or university degree (population share is 41%), and the study was only available in French, excluding non-French speakers (6.3% of Quebec’s population). Data collection was via a telephone computer-assisted interview by three interviewers who “were duly trained and kept exchanging methods of doing throughout the data collection.”^7^ It is disappointing that considerable critical information is lacking such as standard demographic information on socioeconomic status, insurance coverage, health care utilization, prescribing physicians, and geographic location (rural, urban) and without documentation of disease/symptom severity, comorbid illness, or mental health status, all factors that influence the understanding^7^ of treatments. Drug treatment choices for FM have been strongly associated with physician specialty, insurance type, previous medication history, and comorbid disease, information that is lacking in the current report.^12,13^

How can the excessive reported medication use in the study by De Clifford-Faugère et al.^7^ be explained? Participants were asked to name all medications used and prompted to “look in their cabinet or consult their list of medication provided by the pharmacy.” This could have resulted in inclusion of previous over-the-counter or dispensed medications that had been tried but were no longer used. Medications could have been prescribed by different prescribers, although information on prescribers was not collected. Taken together, there was opportunity for overestimation of medication use. Most studies have reported that patients currently use about two medications for FM treatment.^14,15^ In an early study addressing opioid use in 131 patients with FM attending a multidisciplinary pain management unit, patients used an average of 2.3 ±1.3 FM medications.^15^ Similarly, in a more recent survey, of 90 newly referred patients with FM, Lage-Hansen reported a median of two (0, 2) pain medications.^14^ Also, in a large study of 1700 participants enrolled from 58 health care settings in the United States and Puerto Rico and followed longitudinally for 12 months, Robinson et al. reported that most participants were taking two or more medication on most days, but there was a high rate of switching therapy or discontinuation, suggesting that patients continue to search for an appropriate treatment.^16^ In a study of 14,296 patients with FM and 71,324 age and sex-matched controls in Israel, Gendelman et al. reported higher drug use by patients with FM, with more use of nonsteroidal anti-inflammatory drugs and opioids, as reported in the current study, but without stipulating the number of current medications per patient.^8^

In contrast to the excessive medication use in the study by De Clifford-Faugère et al.^7^ numerous authors have reported low adherence to medications by patients with FM, with frequent discontinuation, erratic scheduling, or switching.^9,13,16–18^ In a study of 6626 patients with FM with 72.5% identified as nonadherent, gender, age, race, FM-related comorbidity score, medication type, insurance coverage, and emergency room visits were significant barriers to optimum care and compliance.^17^ Ben-Ami Shor et al. reported low persistence and adherence with FM drug treatments for 3932 newly diagnosed patients with FM, with only 45% dispensed at least one prescription in the year following diagnosis and only 28.8% having filled prescriptions twice in that same year.^9^ There was a high rate of discontinuation of medications, highest for tricyclic antidepressants at 91% and lowest for antidepressants at 74%, and with only 9% identified as very adherent to treatments,^9^ findings that are again dissimilar from the De Clifford-Faugère et al.^7^ study.

Though the study by De Clifford-Faugère et al.^7^ undoubtedly provides a limited snapshot of medication characteristics in FM in a single province in Canada, the results should not be generalizable to the population in general. The excessive use of medication and nonadherence to guideline recommendations is concerning. Either participants reported medication use inaccurately, resulting in overestimation or, alternatively, patients in Quebec are being treated according to a paradigm that is unique and contrary to all current recommendations, which we believe to be unlikely. Or perhaps the thoughts of Osler continue to ring true today. In view of the socioeconomic burden of FM, further studies examining the concordance of FM guideline recommendations with FM management in routine clinical care are urgently needed to understand whether the disparities described in the De Clifford-Faugère et al.^7^ report are more prevalent than previously noted. It is critical that study information be accurate; participants should be representative of the FM population; there should be reliable information on sociodemographic features and comorbidities including mental health status, and past and present treatments should be detailed. We believe that guidelines do represent a reasonable standard of care, and reasons for nonadherence must be identified, understood, and, if possible, rectified.

## References

[1] Wang T, Tan JB, Liu XL, Zhao I. Barriers and enablers to implementing clinical practice guidelines in primary care: an overview of systematic reviews. BMJ open. 2023;13(1):e062158. doi:10.1136/bmjopen-2022-062158. PMC9827241 36609329

[2] Inanici F, Yunus MB. History of fibromyalgia: past to present. Curr Pain Headache Rep. 2004;8(5):369–3. doi:10.1007/s11916-996-0010-6. 15361321

[3] Queiroz LP. Worldwide epidemiology of fibromyalgia. Curr Pain Headache Rep. 2013;17(8):356. doi:10.1007/s11916-013-0356-5. 23801009

[4] Macfarlane GJ, Kronisch C, Dean LE, Atzeni F, Hauser W, Fluss E, Choy E, Kosek E, Amris K, Branco J, et al. EULAR revised recommendations for the management of fibromyalgia. Ann Rheum Dis 2017;76(2):318–28. doi:10.1136/annrheumdis-2016-209724. 27377815

[5] Fitzcharles M-A, Ste-Marie PA, Goldenberg DL, Pereira JX, Abbey S, Choinière M, Ko G, Moulin D, Panopalis P, Proulx J, et al. 2012 Canadian Guidelines for the diagnosis and management of fibromyalgia syndrome. [accessed 2013 May 20]. http://www.canadianpainsociety.ca/pdf/Fibromyalgia_Guidelines_2012.pdf. 10.1155/2013/918216 PMC3673928 23748251

[6] Sommer C, Alten R, Bär KJ, Bernateck M, Brückle W, Friedel E, Henningsen P, Petzke F, Tölle T, Üçeyler N, et al. Drug therapy of fibromyalgia syndrome: updated guidelines 2017 and overview of systematic review articles. Schmerz (Berlin, Germany). 2017;31(3):274–84. doi:10.1007/s00482-017-0207-0. 28493231

[7] De Clifford-Faugère G, Nguena Nguefack H, Godbout-Parent M, Diallo M, Guénette L, Pagé G, Choiniere M, Beaudoin S, Boulanger A, Pinard A, et al. Pain medications used by persons living with fibromyalgia: a comparison between the profile of a Quebec sample and clinical practice guidelines. Can J Pain. 2023. doi:10.1080/24740527.2023.2252037. PMC10653640 38025837

[8] Gendelman O, Shapira R, Tiosano S, Kuntzman Y, Tsur AM, Hakimian A, Comaneshter D, Cohen AD, Buskila D, Amital H. Utilisation of healthcare services and drug consumption in fibromyalgia: a cross-sectional analysis of the Clalit Health Service database. Int J Clin Pract. 2021;75(11):e14729. doi:10.1111/ijcp.14729. 34383362

[9] Ben-Ami Shor D, Weitzman D, Dahan S, Gendelman O, Bar-On Y, Amital D, Shalev V, Chodick G, Adherence AH. Persistence with drug therapy among fibromyalgia patients: data from a large health maintenance organization. J Rheumatol. 2017;44(10):1499–506. doi:10.3899/jrheum.170098. 28765248

[10] Ministère de la Santé et des Services Sociaux. Algorithme de prise en charge de la fibromyalgie. 2021. https://publications.msss.gouv.qc.ca/msss/fichiers/2021/21-947-03W.pdf.

[11] Margolis JM, Princic N, Smith DM, Abraham L, Cappelleri JC, Shah SN, Park PW. Economic impact of adherence to pain treatment guidelines in chronic pain patients. Pain Med. 2019;20(10):1907–18. doi:10.1093/pm/pnz085. 31034040

[12] Robinson RL, Kroenke K, Mease P, Williams DA, Chen Y, D’Souza D, Wohlreich M, McCarberg B. Burden of illness and treatment patterns for patients with fibromyalgia. Pain Med 2012;13(10):1366–76. doi:10.1111/j.1526-4637.2012.01475.x. 22958298

[13] Zhao Y, Chen SY, Wu N, Fraser KA, Boulanger L. Medication adherence and healthcare costs among fibromyalgia patients treated with duloxetine. Pain Pract. 2011;11(4):381–91. doi:10.1111/j.1533-2500.2010.00431.x. 21199311

[14] Lage-Hansen PR, Chrysidis S, Amris K, Fredslund-Andersen S, Christensen R, Ellingsen T. Prevalence of survey-based criteria for fibromyalgia and impact on hospital burden: a 7 year follow-up study from an outpatient clinic. Scand J Rheumatol. 2022:1–10. doi:10.1080/03009742.2022.2145703. 36503382

[15] Fitzcharles MA, Faregh N, Ste-Marie PA, Shir Y. Opioid use in fibromyalgia is associated with negative health related measures in a prospective cohort study. Pain Res Treat. 2013;2013:898493. doi:10.1155/2013/898493. 23577251 PMC3614030

[16] Robinson RL, Kroenke K, Williams DA, Mease P, Chen Y, Faries D, Peng X, Hann D, Wohlreich M, McCarberg B. Longitudinal observation of treatment patterns and outcomes for patients with fibromyalgia: 12-month findings from the reflections study. Pain Med. 2013;14(9):1400–15. doi:10.1111/pme.12168. 23758985

[17] Desai R, Jo A, Marlow NM. Risk for medication nonadherence among medicaid enrollees with Fibromyalgia: development of a validated risk prediction tool. Pain Pract. 2019;19(3):295–302. doi:10.1111/papr.12743. 30369018

[18] Sewitch MJ, Dobkin PL, Bernatsky S, Baron M, Starr M, Cohen M, Fitzcharles MA. Medication non-adherence in women with fibromyalgia. Rheumatology (Oxford). 2004;43(5):648–54. doi:10.1093/rheumatology/keh141. 14983107

